# Radiation-Induced Bystander Effect Mediated by Exosomes Involves the Replication Stress in Recipient Cells

**DOI:** 10.3390/ijms23084169

**Published:** 2022-04-10

**Authors:** Mateusz Smolarz, Łukasz Skoczylas, Marta Gawin, Monika Krzyżowska, Monika Pietrowska, Piotr Widłak

**Affiliations:** 1 Maria Skłodowska-Curie National Research Institute of Oncology, 44-102 Gliwice, Poland; mateusz.smolarz@io.gliwice.pl (M.S.); lukasz.skoczylas@io.gliwice.pl (Ł.S.); marta.gawin@io.gliwice.pl (M.G.); monikaxx41@interia.pl (M.K.); 2 Clinical Research Support Centre, Medical University of Gdańsk, 80-210 Gdańsk, Poland

**Keywords:** bystander effect, exosomes, ionizing radiation, non-targeted effects of radiation, replication stress

## Abstract

Exosomes released by irradiated cells mediate the radiation-induced bystander effect, which is manifested by DNA breaks detected in recipient cells; yet, the specific mechanism responsible for the generation of chromosome lesions remains unclear. In this study, naive FaDu head and neck cancer cells were stimulated with exosomes released by irradiated (a single 2 Gy dose) or mock-irradiated cells. Maximum accumulation of gamma H2A.X foci, a marker of DNA breaks, was detected after one hour of stimulation with exosomes from irradiated donors, the level of which was comparable to the one observed in directly irradiated cells (a weaker wave of the gamma H2A.X foci accumulation was also noted after 23 h of stimulation). Exosomes from irradiated cells, but not from control ones, activated two stress-induced protein kinases: ATM and ATR. Noteworthy is that while direct irradiation activated only ATM, both ATM and ATR were activated by two factors known to induce the replication stress: hydroxyurea and camptothecin (with subsequent phosphorylation of gamma H2A.X). One hour of stimulation with exosomes from irradiated cells suppressed DNA synthesis in recipient cells and resulted in the subsequent nuclear accumulation of RNA:DNA hybrids, which is an indicator of impaired replication. Interestingly, the abovementioned effects were observed before a substantial internalization of exosomes, which may suggest a receptor-mediated mechanism. It was observed that after one hour of stimulation with exosomes from irradiated donors, phosphorylation of several nuclear proteins, including replication factors and regulators of heterochromatin remodeling as well as components of multiple intracellular signaling pathways increased. Hence, we concluded that the bystander effect mediated by exosomes released from irradiated cells involves the replication stress in recipient cells.

## 1. Introduction

The radiation-induced bystander effect (RIBE) is a phenomenon in which non-irradiated cells exhibit several molecular and cellular features typical for a response to ionizing radiation. Such effects, which include changes in gene and protein expression, proliferation, genetic instability, and cell death, are the results of different signals received from nearby (or distant) directly irradiated cells. Clastogenic effects, including DNA strand breaks, chromosome aberrations, and mutations, are the most characteristic features of RIBE. Therefore, it is postulated that local irradiation (e.g., during radiotherapy) may cause systemic cytotoxic and genotoxic damages outside of the radiation field and can even lead to carcinogenic effects beyond the therapy field. Although there is no generally accepted direct evidence yet, it has been suggested that RIBE (and other potential “non-targeted” effects of radiation) may putatively interfere with the results of radiation therapy [1,2,3]. There are several different classes of signals involved in the RIBE. Many “soluble” factors released by irradiated cells to the cell culture media were proposed, including cytokines, chemokines, and other inflammation mediators, as well as reactive oxygen species (ROS), nitric oxide (NO), and different miRNA species [4,5,6]. More recently, extracellular vesicles have also been implicated in this signaling mechanism.

All types of cells released into the extracellular environment different membrane-enclosed vesicles (i.e., extracellular vesicles (EVs)), which appear as key mediators of intercellular communication. Exosomes are the smallest EVs (30–150 nm), which derive from the inward budding of the endosomal membrane to form the multivesicular body that fuses with the plasma membrane to release exosomes to the extracellular space. The cargo of exosomes consists of selected molecules located inside these vesicles or associated with their membrane, which specifically reflects the phenotypic state of donor/parent cells [7,8,9]. The important role of exosomes and other EVs in multiple biological processes initiated a large number of studies focused on their structure and function, which enabled the discovery of different classes of these vesicles with different biogenesis. Therefore, for practical purposes, exosomes and other classes of virus-sized vesicles (<200 nm) are collectively termed small EVs (sEVs) [10]. Endosome-derived exosomes and other classes of sEVs are well-known mediators of cellular response to different types of stress [11,12]. It has been documented that exosomes released by irradiated cells are involved in different aspects of the systemic response to ionizing radiation. It is noteworthy that both cytotoxic/genotoxic and cytoprotective effects were reported in the context of exosome-mediated RIBE. For example, exosomes released by irradiated cells increased levels of chromosomal aberrations and genetic instability in recipient breast cancer cells [13] as well as reduced viability, caused calcium influx, and stimulated production of reactive oxygen species in recipient keratinocytes [14]. On the other hand, exosomes released by irradiated head and neck cancer cells were shown to stimulate DNA repair and enhance the survival of recipient cells subjected to irradiation after exosome uptake [15]. Hence, different effects mediated by exosomes released from irradiated cells could be observed in recipient cells, which putatively depend on the cell context. Moreover, though phenotypic effects associated with such exosomes are readily observed in different experimental models, the molecular mechanism underlying their action remains unclear. Here, we addressed molecular and cellular mechanisms mediated by exosomes released from irradiated cells and found that exosomes induced acute replication stress in recipients, which may have different consequences depending on a specific cellular context.

## 2. Results

**Figure 1:**
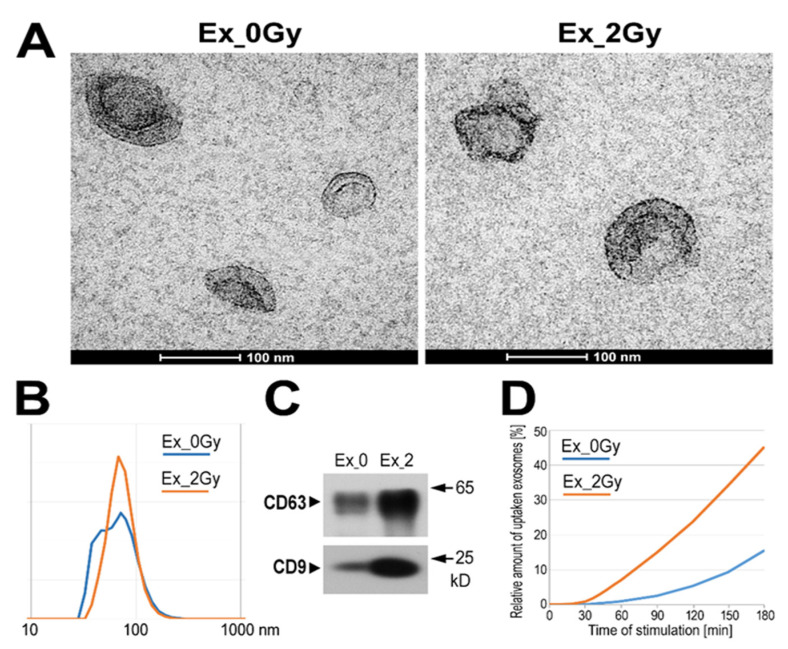
Characteristics of exosomes: TEM imaging of vesicles (**A**); size of vesicles assessed by DLS measurement (**B**); and the presence of exosome markers (**C**) in total sEVs released by mock-irradiated FaDu cells (Ex_0Gy) and cells irradiated with 2 Gy dose (Ex_2Gy); (**D**) relative amounts of internalized vesicles based on the accumulation of exosome membrane-bound dyes (expressed as a percentage of a dye level noted after 6 h of co-incubation).

The total population of sEVs was isolated by size-exclusion chromatography (SEC) from the cell culture media 24 h after irradiation with a single 2 Gy dose or after sham irradiation of FaDu cells (cell line derived from human head and neck carcinoma). The response of FaDu cells to different doses of radiation has previously been analyzed in studies focused on radiation-induced changes in molecular components (i.e., proteins and miRNA) of released exosomes [16,17], which revealed the full viability of cells irradiated with 2 Gy at the time of vesicle collection [17]. Purified vesicles were characterized according to the MISEV2018 guidelines [10]. The morphology and size of the vesicles released either from irradiated cells (Ex_2Gy) or sham-irradiated ones (Ex_0Gy) were analyzed by transmission electron microscopy and dynamic light scattering, which revealed an average vesicle size in the range of 50–100 nm in both cases (Figure 1A,B). The analyzed fraction of vesicles contained typical exosome biomarkers, including CD9 and CD63 (Figure 1C); hence, for simplicity, small EVs present in the studied material are called exosomes hereinafter. We found that irradiated cells released markedly more vesicles, and the amounts of total exosome proteins (TEPs) produced by the same number of cells were at least five-fold higher in the case of Ex_2Gy (which is also shown in Figure 1C). Both types of purified exosomes (i.e., Ex_2Gy and Ex_0Gy) were added to the culture of the naive FaDu cells; the proportion of vesicles and target cells assumed 10-fold excess of (hypothetical) donor cells over recipient cells. To compare the rate of exosome internalization, differently labeled Ex_2Gy and Ex_0Gy were added simultaneously to the culture media. Then, the kinetics of the uptake of a specific dye by cells were analyzed by fluorescence microscopy. We observed that internalization of exosome membrane-bound dye started after 30 min of co-incubation with cells and that internalization of Ex_2Gy-specific dye was 2-3-fold faster than Ex_0Gy-specific dye (Figure 1D; relative uptake of both types of vesicles was normalized regarding their saturation levels after 6 h of co-incubation).

**Figure 2:**
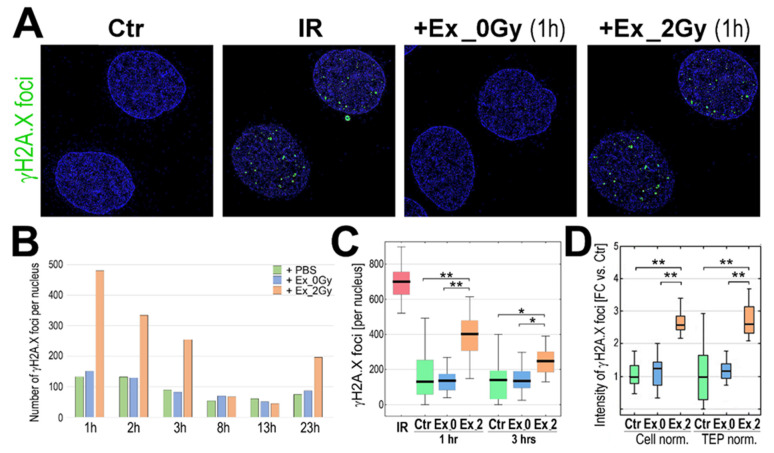
Induction of γH2A.X foci by exosomes from irradiated cells. (**A**) Visualization of γH2A.X foci in FaDu cells co-incubated (1 h) with exosomes released by sham-irradiated (Ex_0Gy) or irradiated (Ex_2Gy) cells; untreated cells (PBS control, Ctr) or cells directly irradiated with 2Gy (IR) were used as controls. (**B**) The number γH2A.X foci after different times of co-incubation with exosomes (1–23 h). (**C**) The number γH2A.X foci after 1 and 3 h of co-incubation with exosomes; directly irradiated cells (IR) were analyzed 1 h after irradiation. (**D**) The relative intensity of γH2A.X foci after 1 h of co-incubation; the number of Ex_0Gy and Ex_2Gy exosomes were normalized according to the number of donor cells (Cell norm.) or according to the total exosome proteins (TEP norm.); the nucleus-integrated intensity was expressed as a fold-change versus PBS-treated controls (FC vs. Ctr). Box plots show the median, minimum, maximum, and lower and upper quartiles; statistically significant differences between groups are represented by asterisks: * *p* < 0.05 and ** *p* < 0.001 (only differences between Ctr and exosome-stimulated cells are shown for clarity).

To address a hypothetical RIBE activated in exosome-stimulated cells, we analyzed the presence of the so-called γH2A.X foci in nuclei of recipient cells, a generally accepted marker of DNA strand breaks which, in the case of radiation-induced DNA double-stranded breaks, showed dose dependence [18]. We found that the levels of γH2A.X foci in the nuclei of cells directly irradiated with a 2 Gy dose (1 h after irradiation) and nuclei of Ex_2Gy-stimulated cells (after 1 h of incubation) were comparably high. In marked contrast, generally low levels of γH2A.X foci in nuclei of Ex_0Gy-stimulated cells were similar to that in the naive untreated control cells (Figure 2A), which indicated that exosomes released by irradiated cells induced RIBE in the naive, non-irradiated cells. The highest level of Ex_2Gy-induced γH2A.X foci was noted after 1 h of co-incubation; then, it gradually decreased, and the levels of γH2A.X foci in cells stimulated with Ex_2Gy were similar to the background level in control cells after 6–8 h of co-incubation. However, another wave of γH2A.X foci was noted after 23 h of stimulation with Ex_2Gy; the latter effect was weaker (Figure 2B), and we further focused on the early effect. The number of γH2A.X foci was significantly higher in cells stimulated with Ex_2Gy than in control naive cells or cells stimulated with Ex_0Gy, both after 1 and 3 h of stimulation (*p* > 0.001 and *p* > 0.05, respectively; Figure 2C). Furthermore, a similar difference between exosomes released by irradiated and mock-irradiated cells was observed when the amounts of Ex_2Gy and Ex_0Gy were standardized according to the actual TEP (Figure 2D), which indicated that the specific effect of exosomes released by irradiated cells was associated with their “quality”, not quantity.

**Figure 3:**
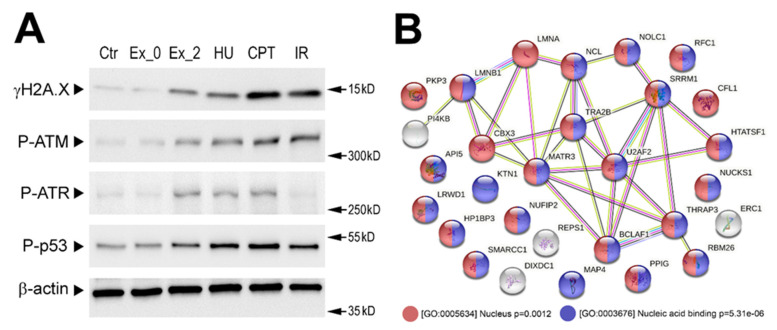
Exosome-stimulated changes in the phosphoproteome of the recipient cells. (**A**) Phosphorylation of selected DNA damage-related proteins analyzed by Western blotting. FaDu cells were stimulated with exosomes released by sham-irradiated (Ex_0Gy) or irradiated (Ex_2Gy) cells, hydroxyurea (HU), and camptothecin (CPT) or directly irradiated with 2 Gy (IR); β-actin was used as a loading control (raw Western blot images are available in Figure S1). (**B**) Network of putative interactions between proteins phosphorylation which was induced in FaDu cells after one hour of stimulation with Ex_2Gy vesicles; color-coded are the proteins associated with two selected GO terms: nuclear localization and nucleic acid binding function (*p*-value refers to the significance of the term overrepresentation).

To search for mechanisms induced by exosomes released by irradiated cells, several molecular features were addressed in the recipient cells. The phosphorylation of H2A.X at Ser-139 could be catalyzed by a few phosphoinositide 3-kinase-related protein kinases including two stress-activated kinases: ATM, activated by radiation-induced double-stranded DNA breaks [19], and ATR, activated in response to single-stranded DNA breaks and during the replication stress [20]. Activation of both protein kinases was analyzed in our experimental model by addressing their active phospho-forms: P-ATM at Ser-1981 and P-ATR at Thr-1989 after one hour of stimulation. We found that stimulation with Ex_2Gy but not with Ex_0Gy resulted in the activation of both ATM and ATR. Of note was that direct irradiation (with 2 Gy) activated primarily ATM (1 h after irradiation). In marked contrast, two factors that are known to induce the replication stress, hydroxyurea (HU) and Topoisomerase I inhibitor camptothecin (CPT), activated both ATM and ATR. All types of stimuli that activated ATM and/or ATR (i.e., Ex_2Gy, IR, HU, and CPT) resulted in phosphorylation of H2A.X at Ser-139 and p53 at Ser-15 (Figure 3A). Hence, one could conclude that stimulation of recipient cells with exosomes released by irradiated cells might induce effects similar to factors known to induce the replication stress.

To further address molecular changes induced by exosomes from irradiated cells, we analyzed changes in the whole phosphoproteome of recipient cells after 1 h of stimulation with exosomes using a shotgun LC-MS approach. The performed analysis revealed 36 phosphopeptides that were significantly upregulated, specifically in cells stimulated with exosomes from irradiated cells (phosphopeptides corresponding to phosphoproteins analyzed in Figure 3A were not identified due to the shortcomings of the untargeted approach). Proteins corresponding to phosphopeptides that were detected in this untargeted approach (Supplementary materials File Table S1) are presented in Figure 3B. In general, upregulated phosphopeptides corresponded to proteins that had nuclear localization (GO:0005634) and nucleic acid binding functions (GO:0003676). Nuclear proteins whose phosphorylation was induced by exosomes from irradiated cells included replication factors (RFC1, RLWD1) and regulators of heterochromatin remodeling (HP1BP3, CBX3, SMARCC1, NCL) as well as components of the nuclear lamina (LMNB1 and LMNA) and nuclear matrix (MATR3, SRRM1). Moreover, proteins whose phosphorylation was specific for cells stimulated with Ex_2Gy included phosphatidylinositol 4-kinase beta (PI4KB, P-Ser-511), a protein involved in the PIP-mediated intracellular signaling network [21], and plakophilin-3 (PKP3, P-Ser-238), a desmosome protein involved in different signaling pathways [22].

**Figure 4:**
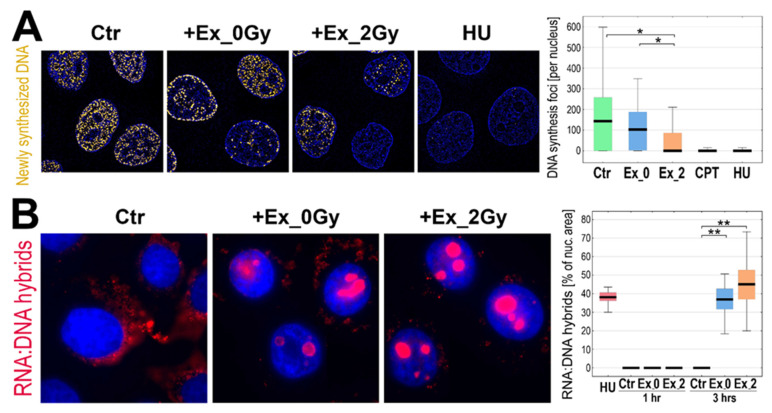
Inhibition of DNA replication by exosomes from irradiated cells. (**A**) Foci of newly replicated DNA initiated in FaDu cells after one-hour of incubation with exosomes released by sham-irradiated (Ex_0Gy) or irradiated (Ex_2Gy) cells or with HU or CPT. (**B**) Visualization of RNA:DNA hybrids in nuclei of FaDu cells incubated for 3 h with Ex_0Gy or Ex_2Gy vesicles; the graph shows the relative occupancy of RNA:DNA hybrids in the nuclei cells incubated with exosomes (1 or 3 h) or HU (1 h). Box plots show the median, minimum, maximum, and lower and upper quartiles; statistically significant differences between groups are represented by asterisks: * *p* < 0.05 and ** *p* < 0.001 (only differences between Ctr and exosome-stimulated cells are shown for clarity).

To verify directly the hypothesis that exosomes from irradiated cells affect DNA replication in recipient cells, the labeled analog of thymidine was added to cells pre-stimulated (1 h) with exosomes (Ex_2Gy or Ex_0Gy) and classical inducers of the replication stress (HU or CPT), and then the foci of newly replicated DNA were visualized by fluorescence microscopy (Figure 4A). Pre-incubation with HU or CPT almost totally inhibited DNA replication. Most interestingly, however, stimulation with Ex_2Gy (but not with Ex_0Gy) also markedly reduced the number of the active replication sites (though the response was more heterogeneous in the cell population). To further address the observed phenomenon, we searched for the presence of RNA:DNA hybrids, the appearance of which is frequently associated with the malfunction of the replication process [23]. We found that after 3 h of stimulation with exosomes, Ex_2Gy in particular, RNA:DNA hybrids were abundant in the nuclei of recipient cells. The nuclear areas occupied by such structures were comparable in cells incubated with HU and stimulated with Ex_2Gy (Figure 4B), which additionally indicated that exosomes released by irradiated cells induced mechanisms resembling the replication stress in the naive recipient cells.

## 3. Discussion

The plethora of phenomena induced in non-irradiated cells by signals from irradiated ones has been known under the collective term of the non-targeted effects of radiation or radiation-induced bystander effect. Several independent mechanisms involved in radiation-induced non-targeted effects exist including two major types of signaling: (1) direct cell-to-cell communication between irradiated and non-irradiated cells through gap junctions; (2) paracrine/endocrine signaling via “soluble” factors secreted by the irradiated cells into the surroundings [4]. In the recent decade, an interesting mediator of these effects was proposed: exosomes and other classes of membrane-enclosed vesicles released by cells into the extracellular space. Several works documented that exosomes from irradiated cells could increase the viability and (radio) resistance of target cells [24,25,26], partly by stimulation of DNA repair in recipients [15], while in other experimental models, exosomes from irradiated cells were clastogenic [27] and stimulated cell death [28]. However, although exosome-mediated mechanisms of the systemic response to radiation may have important clinical implications, many aspects of their action remain unknown. Here, we showed that early exosome-mediated radiation-induced non-targeted effects, observed within one hour of the bystander cell stimulation, included the replication stress in the recipient cell.

Due to the limitations of cytogenetic methods used for the assessment of RIBE, exosome-mediated effects were usually observed in long-term assays [13,14,27,29]. A few exceptions included studies where exosome-mediated RIBE was assessed using molecular tests enabling observation of earlier effects, which could be represented by the report of Arioshi and coworkers, who observed increased accumulation of γH2A.X foci and 53BP1 foci after 24 h of stimulation with exosomes from irradiated cells [30]. However, in other experimental models, where less-defined “soluble” components of conditioned media were tested, the formation of γH2A.X foci and other molecular changes were observed, even after a few minutes of stimulation (with their maximum after 30–60 min) [5]. Such early changes are reported here for the first time in the context of exosome-mediated mechanisms, where a high level of γH2A.X foci accumulated after 60 min of stimulation. Although the second (weaker) wave of γH2A.X foci accumulation was observed after approximately 24 h of stimulation, which corresponded to the effects observed by others, the latter effect was putatively associated with distinct mechanisms induced in recipient cells. Interestingly, molecular changes induced in recipient cells by exosomes from irradiated cells were observed before a substantial number of exosomes were internalized. We started to observe labeled membranes of exosomes inside the target cells after 30 min of co-incubation, while after 60 min of co-incubation, very few exosomes were visible inside the cells (below 5% of the “saturation” level). Moreover, the level of internalized exosomes did not correlate with the extent of changes observed in the recipient cells. This suggested that early exosome-mediated mechanisms of RIBE depended on ligand–receptor interactions between vesicles and the target cells, while the specific molecular cargo delivered to the recipient cells after the exosome uptake could be involved in later events. Hence, it is important to note that phosphatidylinositol 4-kinase beta (PI4KB), a component of the PIP-mediated intracellular signaling network associated with the regulation of cell division [21] and viral replication [31], was rapidly phosphorylated specifically in cells stimulated by exosomes from irradiated cells. 

The generally accepted model assumes that RIBE signals upregulate the generation of reactive oxygen species (ROS) in the targeted cell, which via generation of DNA damage, likely in conjunction with DNA replication and transcription, initiate the DNA damage response (DDR) in the bystander cell. This DDR involves the activation of ATM/ATR-dependent signaling cascades, which aside from participation in the repair of DNA strand breaks, activates Chk1 and Chk2 checkpoint kinases. The activation of checkpoint kinases stops the progression of the cell cycle, which may have either cytoprotective or cytotoxic consequences depending on the cell context [4]. Data presented in this report fit this general model. Reactive oxygen species and ROS-induced DNA damage are known activators of replication stress [32,33]. Incorrect DNA structures that form during the replications stress are recognized by the ATR/ATRIP complexes, which activate further cellular response [3,34]. Here, we observed that early molecular effects induced by exosomes from irradiated cells included activation of both ATM and ATR, which resembled the patterns of ATM/ATR activation observed in cells treated with hydroxyurea and inhibitor of Topo I, i.e., canonical inducers of the replication stress. Indeed, both exosomes from irradiated cells and inducers of the replication stress triggered the suppression of DNA synthesis and subsequent nuclear accumulation of RNA:DNA hybrids. Moreover, the majority of early changes induced by exosomes from irradiated cells in the cellular phosphoproteome involved nuclear proteins associated with replication and the maintenance of chromatin structure. Interestingly, this includes nuclear lamins, which were recently linked to the management of replicative stress [32]. Hence, our data indicated collectively that activation of the replication stress is a key element of the cellular response induced in the recipient cells by exosomes released by irradiated cells. Moreover, proteins phosphorylated specifically in response to exosomes from irradiated cells also included components of desmosome (plakophilin) and cytoskeleton (dixin and MAP4) involved in cell adhesion and migration. This is important to note because previous studies reported that exosomes from irradiated cells increased the motility of recipient cells [35,36].

Hypothetically, three potential explanations for changes induced in bystander cells by vesicles released from irradiated but not from unexposed donor cells could be offered: (i) larger amounts of vesicles released by irradiated donors; (ii) more efficient uptake of vesicles from irradiated donors; (iii) differences in molecular cargo between exosomes from irradiated and non-irradiated cells. Several previous reports [15,25,37], including the current study, documented higher amounts of exosomes recovered from irradiated cells. However, we showed here that if cells were exposed to an equal total exosome protein load, the induction of γH2A.X remained significantly higher after the stimulation with vesicles from irradiated cells, which indicated that the differences between exosomes from irradiated and non-irradiated donors were mostly qualitative, not only quantitative. It has been reported that radiation affects the cellular uptake of exosomes: irradiation can enhance the uptake of exosomes by affected cells [15,38], and exosomes from irradiated cells could be taken faster by recipient cells [35]; the latter effect was observed also in our study. However, as discussed above, the early effect of exosomes seemed to be independent of their uptake into the target cell. Finally, numerous papers showed that radiation globally changed the protein and RNA content of released exosomes [16,17,36,39,40], and functional changes in bystander cells could be attributed to protein and RNA (mRNA, miRNA, and lncRNA) cargo delivered by exosomes [24,26,27,36]. More recently, mitochondrial DNA transferred by exosomes was proposed as another signal involved in the RIBE [30]. Importantly, however, our data discussed above suggested that ligand-receptor interactions were important for early effects induced in a target cell by exosomes released by irradiated donors. Hence, radiation-upregulated proteins that putatively localize in exosome membranes are of particular interest. In this context, it is important to note that about half of proteins characteristic for exosomes released by irradiated FaDu cells were associated with the GO term “membranes” [16]. Similarly, the third part of proteins, the level of which increased after irradiation in exosomes released by another head and neck cancer cell line UM-SCC6, was associated with the GO term “plasma membrane” including RAC1 and RAC2 membrane-associated small GTPases that augment the production of ROS by NADPH oxidase [40]. Therefore, the potential role of radiation-upregulated proteins with actual exosome membrane localization in DDR signaling (the generation of ROS in particular) is an interesting subject of future studies.

## 4. Materials and Methods

### 4.1. Cell Model

The FaDu cell line (HTB-43) was purchased from ATCC (as a component of the Head and Neck Cancer Panel; TCP-1012); these cells were originally derived from human squamous cell carcinoma located in the hypopharynx (HPV negative). Cells were grown in a modified MEM medium with a final concentration of non-heat-inactivated FBS (Thermo Fisher Scientific, Waltham, MA, USA; 10270106) of 10% (*v*/*v*) as described in detail elsewhere [17]. The medium was replaced 3 times per week, and cells were incubated at 37 °C, in air with 5% CO_2_. At the time of the experiments, cells were between passages 10 and 15. Cells were irradiated with a single dose of 2 Gy at a dose rate of 1 Gy/min using 6 MeV photons and a linear accelerator (True Beam, Varian, Palo Alto, CA, USA). To analyze the effects of exosomes, naive cells were incubated with sEVs purified from cell culture media (details below) on a cell imaging cover glass (Eppendorf, Hamburg, Germany; 0030742036) coated with poly-L-lysine (Merck, Darmstadt, Germany; P4832) before seeding the cells. The ratio of vesicles to recipient cells was calculated based on the number of sEV-releasing cells (donor cells to recipient cells ratio of 10:1). Recipient cells were incubated with sEVs in a fresh medium containing 5% (*v*/*v*) Gibco exosome-depleted FBS (Thermo Fisher Scientific, Waltham, MA, USA; A2720801). Moreover, when indicated, cells were incubated with hydroxyurea or camptothecin at a final concentration of 2 mM and 2.5 µM, respectively.

### 4.2. Purification and Characterization of sEVs 

For vesicle isolation, FaDu cells were grown in T175 flasks (Greiner BioOne, Kremsmünster, Austria; 660175) in a modified MEM with a final concentration of non-heat-inactivated FBS (Thermo Fisher Scientific, Waltham, MA, USA; 10270106) of 10% (*v*/*v*). The standard culture medium was replaced with a fresh one containing 5% (*v*/*v*) Gibco Exosome-Depleted FBS (Thermo Fisher Scientific, Waltham, MA, USA; A2720801). Then, cells were irradiated (or sham-irradiated) and the cell culture medium was harvested 24 h later. Forty milliliters of medium (corresponding to approximately 2.7 × 10^7^ cells) were centrifuged sequentially at 200× *g* (10 min), 2000× *g* (10 min), and 10,000× *g* (30 min) to remove cellular debris and then filtered with a 0.22 μm filter (Merck, Darmstadt, Germany; SLGP033RB) to remove large EVs (including putative apoptotic bodies or microvesicles). The filtered medium was concentrated to 1 mL using a Vivacell100 ultrafiltration unit (Sartorius, Göttingen, Germany; VC1042) and then loaded onto an Econo-Pac 10DG column (BioRad, Hercules, CA, USA; 732-2010) filled with 10 mL of Sepharose CL-2B (GE Healthcare, Chicago, IL, USA; 17014001) at a length of 6 cm. The column was left until dripping ceased (void volume); then, 1 mL fractions were eluted by loading stepwise 1 mL of PBS; sEVs of interest were eluted in fraction 4 (F4). For further analyses, 1 mL of fraction F4 was concentrated to approximately 50 µL using Vivaspin500 ultrafiltration tubes (Sartorius, Göttingen, Germany; VS0102). Exosome markers (CD9, CD63) were analyzed by Western blot as described in detail elsewhere [41]. The size distribution profile of EVs was estimated by the dynamic light scattering (DLS) measurement using a Zetasizer Nano-ZS90 instrument (Malvern Instruments, Malvern, UK) as described in detail elsewhere [41]. To assess the morphology of the vesicles, transmission electron microscopy (TEM) analysis was performed according to the protocol provided by Thery et al. [42] as described in detail elsewhere [41].

### 4.3. Western Blot Analysis

Whole-cell lysates were prepared in the RIPA buffer (50 mM Tris-HCl, pH 8.0, 150 mM NaCl, 1.0% NP-40, 0.5% sodium deoxycholate, and 0.1% sodium dodecyl sulfate) enriched with protease (Roche, Mannheim, Germany; 11836153001) and phosphatase (Roche, Mannheim, Germany; 04906845001) inhibitors. The concentration of proteins in the analyzed samples was assessed using the Pierce^TM^ BCA Protein Assay kit (Thermo Fisher Scientific, Waltham, MA, USA; 23225) according to the manufacturer’s instructions. Proteins samples (15 µg) were mixed with the loading buffer to a final concentration of 2% (*v*/*v*) SDS, 0.1% (*v*/*v*) bromophenol blue, 10% (*v*/*v*) glycerol, and 100 mM DTT, then denatured for 5 min at 95 °C and separated by 12% SDS–polyacrylamide gel electrophoresis followed by wet transfer onto nitrocellulose membranes (Thermo Fisher Scientific, Waltham, MA, USA; 88018). Membranes were blocked for 1 h in 5% non-fatty milk and 0.1% Tween in PBS, and then the primary antibodies were added for 16 h incubation at 4 °C: anti-P-Ser15-p53 (Cell Signaling Technology, Danvers, MA, USA, 9284S; 1:1250), anti-P-Ser139-H2A.X (Cell Signaling Technology, 9718S; 1:1000), anti-P-Ser1981-ATM (Cell Signaling Technology, 5883S; 1:1250), anti-P-Thr1989-ATR (Thermo Fisher, PA-5-77873; 1:1000), and anti-β-Actin (Cell Signaling Technology, 4967S; 1:1000). After triplicate washes, a secondary antibody conjugated with HRP was added for 1 h at 23 °C. Chemiluminescence detection of bands was performed with WesternBright Sirius HRP substrate (Advansta, San Jose, CA, USA, K-12043-D10) according to the manufacturer’s instructions. 

### 4.4. Immunocytochemistry and Fluorescence Microscopy

Cells co-incubated with sEVs were washed three times with PBS and fixed with 4% formaldehyde solution in PBS for 20 min. After three washes with PBS, cells were permeabilized in 0.1% Triton X-100/0.1× citrate buffer for 5 min on ice and washed three times with PBS. Non-specific binding of antibody was blocked with a 3% BSA solution in PBS for 30 min at 23 °C. The preparations were incubated with the primary antibodies anti-P-Ser139-H2A.X (Cell Signaling Technology, Danvers, MA, USA, 9718S; 1:400) or anti-DNA-RNA hybrid S9.6 (Kerafast, Boston, MA, USA, ENH001; 1:100) in 3% BSA/PBS for 1 h and then with the secondary antibody conjugated with FITC (2% BSA/PBS) for 1 h in the dark at 23 °C. In order to identify newly replicated DNA after stimulation of sEV cells, incubation was performed for 30 min with a thymine analog EdU (5-ethynyl-2′-deoxyuridine; Thermo Fisher Scientific, Waltham, MA, USA; C10339) at a final concentration of 10 µM. Then, the cells were washed three times with PBS and fixed with 4% formaldehyde in PBS for 20 min. Nuclei were counterstained with DAPI, and the preparations were examined using the ELYRA 7 system (Carl Zeiss, Oberkochen, Germany) at a magnification of 63×. To visualize exosome uptake, purified vesicles were stained with PKH26 (Ex_0Gy) or with PKH67 (Ex_2Gy) (Merck, Darmstadt, Germany; MINI26 and MINI67, respectively) according to the manufacturer’s procedure. Then, the excess dye was removed using Exospin 3 kDa columns (Thermo Fisher Scientific, Waltham, MA, USA; 4484449). The stained vesicles (both types simultaneously) were added to the FaDu cells and a 24 h real-time observation was performed using the ELYRA 7 system at a magnification of 20×. 

### 4.5. Protein Identification by LC-MS/MS

Whole-cell lysates in the RIPA buffer were prepared for mass spectrometry-based shotgun global phosphoproteomic analysis according to Supplementary Protocol P1 given in the Supplementary Materials. Enrichment of phosphopeptides was realized using titanium dioxide according to the protocol of Borisova et al. [43] with modifications. Peptides were analyzed using the Dionex UltiMate 3000 RSLC nanoLC System coupled with the Q Exactive Plus Orbitrap mass spectrometer (Thermo Fisher Scientific). The spectrometer was operated in data-dependent MS/MS mode with survey scans acquired at the resolution of 70,000 at *m/z* 50 in the MS mode, and 17,500 at *m/z* 200 in the MS2 mode. Based on the Swiss-Prot human database, peptide and fragment ion masses were used for protein identification with a precision tolerance of 10 ppm and 0.02 Da, respectively. A protein was considered as positively identified if at least one specific peptide was detected, and the peptide score met the significance threshold FDR = 0.01. Protein abundances were determined in Proteome Discoverer by using the Precursor Ions Area detector mode, which uses an average intensity of the three most intensive peptides for a given protein, normalized to the total ion current (TIC). A detailed description of the implemented protocol is provided in Supplementary Protocol P2. The obtained data were deposited to the ProteomeXchange Consortium [44] via the PRIDE [45] partner repository with the data set identifier PXD032143.

### 4.6. Statistical and Bioinformatics Analyses

The significance of the differences between the analyzed groups was assessed using the Kruskal–Wallis test followed by Dunn’s post hoc test for pairwise comparisons; *p* < 0.05 was found to be statistically significant. To assess the effects of exosome stimulation on phosphoproteome of the recipient cells, nine ratios between 3 technical replicas of pairwise compared samples (Ex_0Gy vs. Ex_2Gy) were established, and their global distributions were modeled by Gaussian mixture allowing for quantification of differences between the compared samples [46]. The major components located around the ratio 1.0 were considered as the model of the “not changed” feature, while thresholds for “changed” features were set at 1.710. A feature was considered upregulated (or downregulated) when the median fold-change ratio for all combinations of replicas exceeded the abovementioned thresholds. The String-db knowledgebase [47] was used to predict potential interactions between selected proteins (accessed on 25 January 2022).

## Data Availability

The mass spectrometry proteomics data were deposited at the ProteomeXchange Consortium with the data set identifier: PXD032143.

## Supplementary Materials

The following supporting information can be downloaded at: https://www.mdpi.com/article/10.3390/ijms23084169/s1.
