# Correction: Smolarz et al. Radiation-Induced Bystander Effect Mediated by Exosomes Involves the Replication Stress in Recipient Cells. *Int. J. Mol. Sci.* 2022, *23*, 4169

**DOI:** 10.3390/ijms27073254

**Published:** 2026-04-03

**Authors:** Mateusz Smolarz, Łukasz Skoczylas, Marta Gawin, Monika Krzyżowska, Monika Pietrowska, Piotr Widłak

**Affiliations:** 1 Maria Skłodowska-Curie National Research Institute of Oncology, 44-102 Gliwice, Poland; mateusz.smolarz@io.gliwice.pl (M.S.); monika.pietrowska@io.gliwice.pl (M.P.); 2 2nd Department of Radiology, Medical University of Gdańsk, 80-210 Gdańsk, Poland

In the original publication [1], there was a mistake in Figure 2 as published. In panel A of Figure 2, the small representative inset intended to illustrate the response of cells incubated with exosomes released by irradiated cells (Ex_2Gy) was mistakenly taken from an incorrect subfolder containing insets of irradiated cells. Following correction, the appropriate representative inset derived from the correct micrograph illustrating cells incubated with exosomes released by irradiated cells was used to prepare the revised version of Figure 2A. The corrected Figure 2 and caption appear below. The authors state that the scientific conclusions are unaffected. This correction was approved by the Academic Editor. The original publication has also been updated.

## Figures and Tables

**Figure 2:**
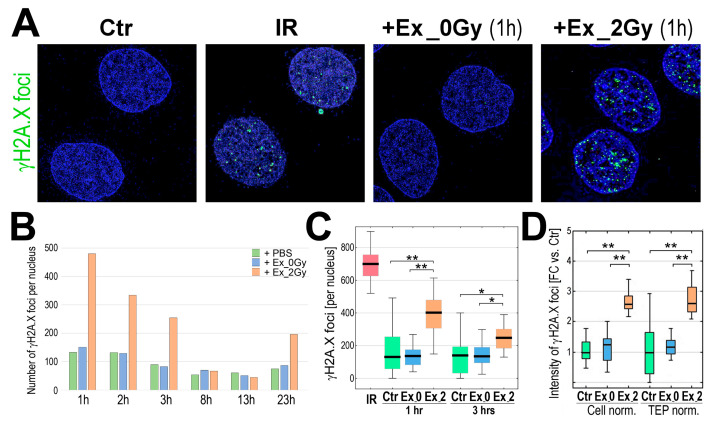
Induction of γH2A.X foci by exosomes from irradiated cells. (**A**) Visualization of γH2A.X foci in FaDu cells co-incubated (1 h) with exosomes released by sham-irradiated (Ex_0Gy) or irradiated (Ex_2Gy) cells; untreated cells (PBS control, Ctr) or cells directly irradiated with 2Gy (IR) were used as controls. (**B**) The number γH2A.X foci after different times of co-incubation with exosomes (1–23 h). (**C**) The number γH2A.X foci after 1 and 3 h of co-incubation with exosomes; directly irradiated cells (IR) were analyzed 1 h after irradiation. (**D**) The relative intensity of γH2A.X foci after 1 h of co-incubation; the amounts of Ex_0Gy and Ex_2Gy exosomes were normalized according to the number of donor cells (Cell norm.) or according to the Total Exosome Proteins (TEP norm.); the nucleus-integrated intensity was expressed as a fold-change versus PBS-treated controls (FC vs. Ctr). Box plots show the median, minimum, maximum, lower, and upper quartile; statistically significant differences between groups are represented by asterisks: (*) = *p* < 0.05, (**) = *p* < 0.001 (only differences between Ctr and exosome-stimulated cells are shown for clarity).
