# Supplementary material for: Radiation-Induced Bystander Effect Mediated by Exosomes Involves the Replication Stress in Recipient Cells
Source: Int J Mol Sci. 2022 Apr 10;23(8):4169. doi: 10.3390/ijms23084169 (PMC9029583; doi:10.3390/ijms23084169)
Supplement: Supplementary file 1 [file ijms-23-04169-s001.zip › Supplementary-Protocols-P1-P2.pdf]

Supplementary Protocol P1.

Enrichment of phosphorylated peptides for MS-based analysis.

1. Lysates in RIPA buffer (300 µg of total protein extract in each) were subjected to protein precipitation with four volumes of ice-cold acetone and kept at -20 °C overnight, then centrifuged (20'000 RCF, 30 min, 4°C).
2. Protein pellets were dissolved in 1 mL 8M urea in 50 mM NH_4HCO_3 and subjected to in-solution digestion with Trypsin/Lys-C Mix (Promega) with the enzyme to protein ratio of 1:50 (m/m). Enzymatic digestion was preceded with reduction of disulfide bridges using dithiothreitol (final concentration of DDT: 5 mM) and alkylation of cysteines with iodoacetamide (final concentration of IAA: 15 mM).
3. The obtained protein digests were purified using solid-phase extraction on octadecyl bed (Macherey Nagel Chromabond C18, 100 mg/ 1mL cartridges), the collected eluates were mixed with acetonitrile and trifluoroacetic acid to obtain the final concentration of ACN and TFA of 50% and 6%, respectively.
4. Peptide samples were subjected to phosphopeptide enrichment using metal oxide affinity chromatography (MOAC) with titanium dioxide as a sorbent. Two TopTips (Glygen) filled with porous TiO_2 (bed volume of 15 µL) were utilized for each sample to perform two rounds of enrichment and gain higher recovery of phosphopeptides from each sample.
5. Phosphopeptide sorption was realized in a batch mode as follows: a portion of TiO_2 sorbent contained in one TopTip was transferred to a 2 mL Eppendorf tube and preconditioned in the Binding Buffer (50% ACN, 6% TFA) upon vigorous mixing using a laboratory vertical rotator, then spun shortly. The activated sorbent was subsequently soaked with a sample solution and incubated at room temperature for 1 hour upon vigorous mixing. Then, the sample was transferred to the second portion of activated TiO_2 beads and incubated for another hour upon mixing.
6. Both portions of TiO_2 were washed twice with the Wash Buffer (50% ACN, 0.1% TFA), the beads and the second portion of the Wash Buffer were transferred back to the emptied TopTips and the retained phosphopeptides were released from each portion of the bed in a two-step elution: first with 0.1 mL of the Elution Buffer 1 (5% NH_3 aq), then with 0.1 mL of the Elution Buffer 2 (10% NH_3 aq, 25% ACN); each elution step was aided with centrifugation at 400 RCF, 2 min.
7. Eluates obtained from both portions of TiO_2 were subsequently merged and the volatile constituents (i.e., NH_3 and ACN) were removed via vacuum evaporation.
8. All samples were acidified with TFA and desalted using StageTips filled with 8 pieces of octadecyl disk (C18 disks, Empore).
9. Eluates were dried out in a vacuum centrifuge and peptides were reconstituted in 20 µL of water.
10. Peptide content in each sample was assessed using the tryptophan fluorescence method.

Supplementary Protocol P2.

LC-MS analysis of phosphorylated peptides.

1. LC-MS/MS analysis of phosphopeptide samples was carried out using the Dionex UltiMate 3000 RSLC nanoLC System in conjunction with the Q Exactive Plus Orbitrap mass spectrometer (Thermo Fisher Scientific). Peptides from each phosphopeptide-enriched sample (1.0 µg) were separated on a reverse-phase Acclaim PepMap RSLC nanoViper C18 column (75 µm × 25 cm, 2 µm granulation) using 0.1% FA in LC-MS grade water as a Mobile Phase A and 80% acetonitrile with 0.1% FA in LC-MS grade water as a Mobile Phase B at 30°C and a flow rate of 300 nL/min (for 120 minutes).
2. For additional desalting purposes samples were loaded onto a C18 trap column for 3 minutes using 0.1% FA in LC-MS grade water as a Loading Buffer. After desalting the trap column was switched with the analytical column and peptides were eluted with the binary gradients of 3-8% of the Mobile Phase B for 7 min, 8-28% Mobile Phase B for 50 min, 28-50% Mobile Phase B for 20 min and 50-80% Mobile Phase B in a further 5 min. Finally, rinsing off the column in 80% Mobile Phase B for 15 min and equilibrating in 3% Mobile Phase B for 20 min was performed.
3. The spectrometer was operated in data-dependent MS/MS mode with survey scans acquired at the resolution of 70,000 at m/z 50 in MS mode, and 17,500 at m/z 200 in MS2 mode. Spectra were recorded via positive ion scanning mode in the range of 380-1500 m/z. Using normalized collision energy of 28, higher-energy collisional dissociation (HCD) was used to fragment ions.
4. Based on the Swiss-Prot human database (release 2021_03 containing 20 325 sequence entries, 11 364 617 residues), peptide and fragment ion masses were used for protein identification with a precision tolerance of 10 ppm and 0.02 Da, respectively. Cysteine carbamidomethylation was set as a fixed modification, while methionine oxidation and phosphorylation of serine, threonine, tyrosine, histidine, and aspartic acid were chosen as variables modifications.
5. All raw data derived from each dataset were imported into Protein Discoverer v. 2.3.0 (Thermo Fisher Scientific) <Thermo raw files> to identify and quantify proteins (Sequest engine was used to search the database). Using the search engine, proteins were considered as positively identified if at least one peptide per protein was detected by the search engine, and the peptide score met the significance threshold FDR = 0.01 (based on the Percolator algorithm); a protein was also considered as “present” if detected in at least one sample of a given type.
6. Protein abundances were determined in Proteome Discoverer by using the Precursor Ions Area detector mode, which uses an average intensity of three most intensive peptides for a given protein, normalized to the total ion current (TIC).
