# Supplementary figures and images for: Radiation-Induced Bystander Effect Mediated by Exosomes Involves the Replication Stress in Recipient Cells
Source: Int J Mol Sci. 2022 Apr 10;23(8):4169. doi: 10.3390/ijms23084169 (PMC9029583; doi:10.3390/ijms23084169)

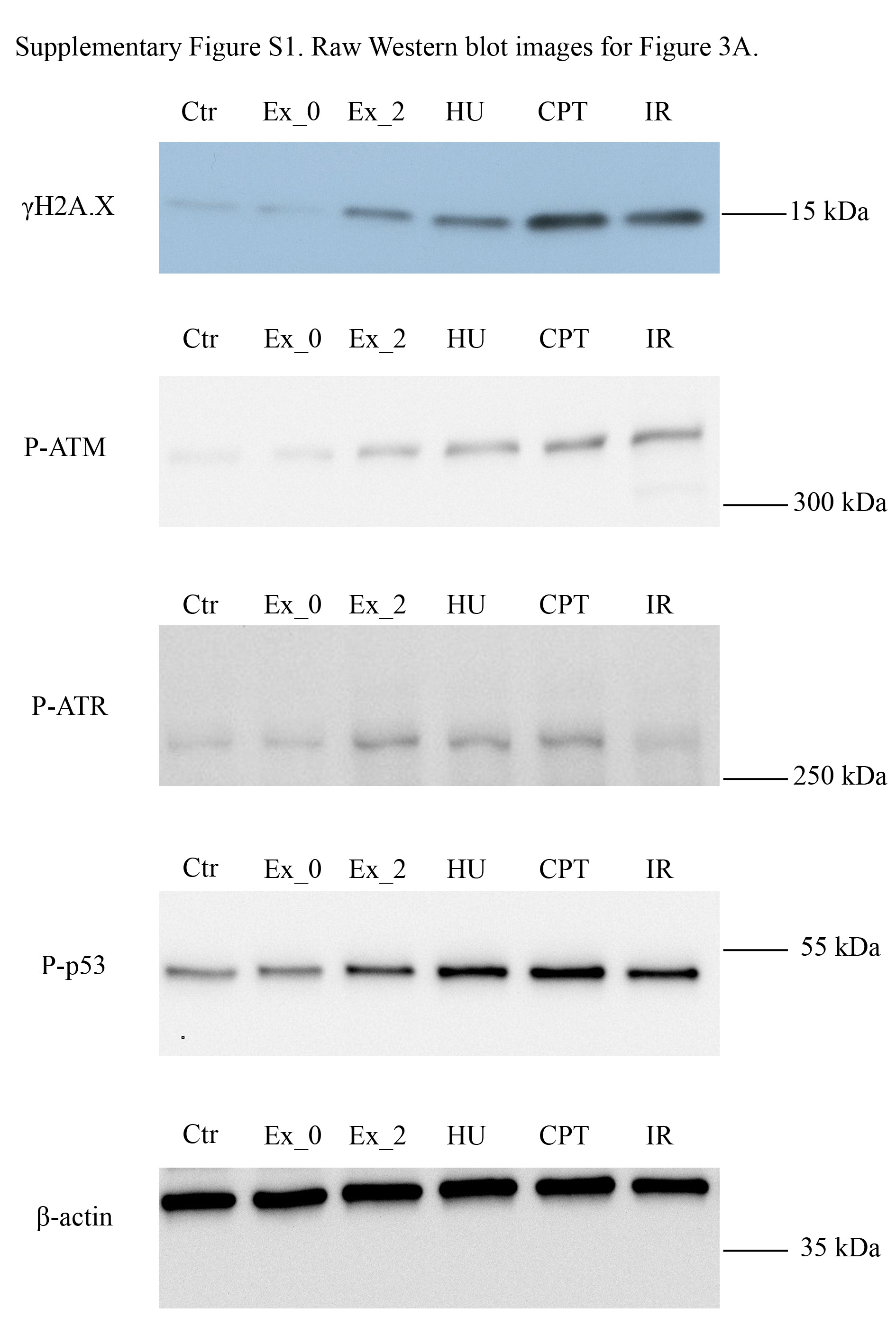

Supplement: Supplementary file 1 [file ijms-23-04169-s001.zip › Supplentary Figure S1.jpg]
